# The complete mitochondrial genome sequence of *Aquarius elongatus* (Hemiptera: Gerridae)

**DOI:** 10.1080/23802359.2021.1895001

**Published:** 2021-03-18

**Authors:** Ying Cui, Hongxia Yang, Xiaoxuan Tian

**Affiliations:** State Key Laboratory of Component-Based Chinese Medicine, Tianjin University of Traditional Chinese Medicine, Tianjin, China

**Keywords:** *Aquarius elongatus*, mitochondrial genome

## Abstract

In this study, we report the complete mitochondrial genome of *Aquarius elongatus*. The mitogenome was 15,370 bp in length, comprising 13 protein-coding genes, 22 tRNA genes, 2 rRNA genes, and a control region. Maximum-likelihood (ML) phylogenetic tree indicated that *Aquarius elongatus* has a close relationship with *Aquarius paludum*. In general, this study provides meaningful genetic information for *A. elongatus.*

*Aquarius elongatus,* also known as the Giant water strider, is a common aquatic insect of the Order Hemiptera, family Gerridae. This family comprises eighty genera and approximately 700 described species (Damgaard 2006). Due to their general high abundance in many freshwater systems, their unique morphological and their two-dimensional habitat have been in the focus of entomological and ecological research for a long time (Damgaard and Cognato 2005; Havemann et al. 2018). The present study is an attempt to improve our molecular knowledge about *A. elongatus* and it is necessary to obtain the whole gene sequences of mitogenome for phylogenetic studies.

Adult specimens of *A. elongatus* were collected by insect net from Kunming city, Yunnan Province (102.73° E, 25.04° N), China. The collected specimens were quickly frozen in liquid nitrogen and then stored at −80 °C until DNA extraction. The voucher specimens were deposited in the State Key Laboratory of Component-Based Chinese Medicine, Tianjin University of Traditional Chinese Medicine (Ying Cui, CQL8179270@126.com), under the voucher number of A20060701. Total genomic DNA was extracted from muscle tissue of thorax by a CTAB-based method (Reineke et al. 1998). The total length of *A. elongatus* mtDNA was amplified by polymerase chain reaction (PCR). It was performed with TaKaRa LA PCR Kit Ver. 2.1 according to the manufacturer’s recommendations. The primers used in this study were listed in Supplementary Table S1. The PCR products were electrophoresed in 0.7–1% agarose gel and then purified. All the products were sequenced directly by various commercial biological companies in double direction by primer walking. BioEdit (Hall 1999) was employed to assemble sequences. Protein-coding region was identified by Open Reading Frame Finder (ORF Finder) provided by NCBI (http://www.ncbi.nlm.nih.gov/gorf/gorf.html) with invertebrate mitochondrial genetic codes. Ribosomal RNA genes were assumed to extend to the boundaries of flanking genes and also compared with other hemipteran mitochondrial sequences by CLUSTAL X 1.83 (Thompson 1997). Transfer RNA analysis was conducted using the tRNAscan-SE Web Server (Lowe and Chan 2016). Finally, the complete mitogenome was submitted to GenBank under accession number KR920101.

The mitogenome of *A. elongatus* was 15,370 bp in length, including a control region. Additionally, a total of 37 genes were annotated, including 13 protein-coding genes, 22 tRNA genes, and two rRNA genes. The nucleotide base composition was 44.3% A, 14.7% C, 9.8% G and 31.2% T, the overall GC content was 24.5%. Overall, the mitogenome features of *A. elongatus* were consistent with other heteropterans that have reported in terms of genomic structure (Hua et al. 2009).

To investigate the phylogenetic position of *A. elongatus* in family Gerrinae, *A. elongatus* and other seven Gerridae species were selected to construct a phylogenetic tree. We extracted the nucleotide sequences of shared PCGs from these mitogenomes. The 13 shared PCGs were aligned individually using PhyloSuite_v1.1.15 (Zhang et al. 2020), and all aligned PCGs were then concatenated. Maximum likelihood (ML) analysis was performed using IQ-TREE (Nguyen et al. 2015) (Figure 1) under the model automatically selected. As expected, the result indicated that *A. elongatus* had a close relationship with *A. paludum*.

**Figure 1.**
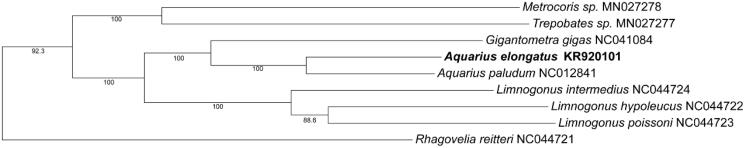
Maximum-likelihood tree inferred from complete mitochondrial genome sequences of Gerridae. *Rhagovelia reitteri* were set as the outgroup. The numbers above the lines were bootstrap support values for ML analyses.

## Data Availability

The genome sequence data that support the findings of this study are openly available in GenBank of NCBI at (https://www.ncbi.nlm.nih.gov/) under the accession no. KR920101.
